# Validation of reference genes for quantitative real-time PCR (qPCR) analysis of *Actinobacillus suis*

**DOI:** 10.1186/s13104-015-1045-8

**Published:** 2015-03-18

**Authors:** Adina R Bujold, Janet I MacInnes

**Affiliations:** Department of Pathobiology, Ontario Veterinary College, University of Guelph, 50 Stone Road East, N1G 2W1 Guelph, Ontario Canada

**Keywords:** qPCR, Reference gene, Pasteurellaceae

## Abstract

**Background:**

Quantitative real-time PCR is a valuable tool for evaluating bacterial gene expression. However, in order to make best use of this method, endogenous reference genes for expression data normalisation must first be identified by carefully validating the stability of expression under experimental conditions. Therefore, the objective of this study was to validate eight reference genes of the opportunistic swine pathogen, *Actinobacillus suis*, grown in aerobic cultures with (Epinephrine) or without (Aerobic) epinephrine in the growth medium and in anoxic static cultures (Anoxic), and sampled during exponential and stationary phases.

**Results:**

Using the RefFinder tool, expression data were analysed to determine whether comprehensive stability rankings of selected reference genes varied with experimental design. When comparing Aerobic and Epinephrine cultures by growth phase, *pyk* and *rpoB* were both among the most stably expressed genes, but when analysing both growth phases together, only *pyk* remained in the top three rankings. When comparing Aerobic and Anoxic samples, *proS* ranked among the most stable genes in exponential and stationary phase data sets as well as in combined rankings. When analysing the Aerobic, Epinephrine, and Anoxic samples together, only *gyrA* ranked consistently among the top three most stably expressed genes during exponential and stationary growth as well as in combined rankings; the *rho* gene ranked as least stably expressed gene in this data set.

**Conclusions:**

Reference gene stability should be carefully assessed with the design of the experiment in mind. In this study, even the commonly used reference gene *16S rRNA* demonstrated large variability in stability depending on the conditions studied and how the data were analysed. As previously suggested, the best approach may be to use a geometric mean of multiple genes to normalise qPCR results. As researchers continue to validate reference genes for various organisms in multiple growth conditions and sampling time points, it may be possible to make informed predictions as to which genes may be most suitable to validate for a given experimental design, but in the meantime, the reference genes used to normalise qPCR data should be selected with caution.

## Background

*Actinobacillus suis* is a Gram negative facultative anaerobe which is a frequent member of the normal microbiome of swine tonsils of the soft palate [1]. It is also an important pathogen in pigs of all ages, where it can cause septicaemia and sequelae such as meningitis, arthritis, and pleuropneumonia [2]. However, little is known about the pathogenesis of *A. suis*, including the expression of virulence-associated genes.

Quantitative real-time PCR (qPCR) is a sensitive method for the determination of bacterial gene expression. In most qPCR studies, endogenous reference genes are used to control for sample-to-sample variations that may arise due to differences in cell number and efficiency of RNA extraction and cDNA synthesis, among other factors [3]. Further, using a reference gene permits for normalisation of multiple genes to a common control, allowing for more robust data comparison. However, several recent studies suggest that rather than relying on commonly used genes, reference genes should be carefully selected and rigorously validated [3-6]. Also, it has been suggested that using the geometric mean of data collected from multiple reference genes is more appropriate than relying on a single reference gene for normalisation [7].

Therefore, the objective of this work was to validate reference genes of a clinical isolate of *A. suis*, H91-0380, grown in different conditions and sampled during different growth phases. Eight reference genes were selected for evaluation based on published expression studies of other members of the family *Pasteurellaceae* [8,9] and the analysis of the *A. suis* genome for the presence of commonly used reference genes.

## Methods

### Bacterial strains and growth media

*Actinobacillus suis* H91-0380, a virulent O2:K2 clinical isolate collected in Southwestern Ontario, Canada, from a pig with septicaemia [10,11] (Table 1), was grown in brain heart infusion (BHI) (BD, Sparks, MD); epinephrine (Sigma-Aldrich, St. Louis, MO) was added to the growth medium at the time of inoculation to a final concentration of 50 μM.

**Table 1:** **Bacterial strain and genes used in this work**

**Bacterial strains or genes**	**Characteristic(s)**	**Reference or locus tag**
Strain		
*Actinobacillus suis* H91-0380	O2:K2 clinical isolate	[10,11]
Genes		
*16S rRNA*	16S ribosomal subunit	ASU2_r11469, ASU2_r11471, ASU2_r11473, ASU2_r11475, ASU2_r11477, ASU2_r11479
*ackA*	Acetate kinase A	ASU2_03825
*glyA*	Glycine/serine hydroxymethyltransferase	ASU2_01625
*gyrA*	DNA gyrase subunit A	ASU2_01490
*proS*	Prolyl-tRNAsynthetase	ASU2_08190
*pyk*	Pyruvate kinase	ASU2_06045
*rho*	Transcription termination factor Rho	ASU2_01275
*rpoB*	DNA-directed RNA polymerase subunit β	ASU2_09775

### Growth conditions

Aerobic cultures of *A. suis* H91-0380 (+/− epinephrine) were grown in BHI at 37°C with shaking at 200 rpm. Anoxic static cultures were grown without shaking in BHI at 37°C + 5% CO_2_ in 1 mL aliquots in sealed 1.5 mL microcentrifuge tubes.

Growth curves were done in triplicate by measuring the OD_600_ of *A. suis* every 30 minutes from the time of inoculation until stationary phase was achieved, and then three or more additional times. Sampling time points for early exponential and early stationary phases of growth were determined, and the number of CFU/mL was calculated by plating 10-fold serial dilutions of the cultures on Columbia agar with 5% sheep blood (Oxoid Co., Nepean, ON).

### RNA extraction

Samples of ~1×10^8^ CFU were collected from aerobic cultures at 60 and 180 minutes post-inoculation (mpi), and from anoxic cultures at 60 and 210 mpi (representing exponential and stationary phases, respectively). Cells were pelleted at 6000 × g for 5 minutes at 4°C and the supernatant was decanted. Cells were lysed as previously described [12]. Briefly, the pellet was suspended in 100 μL pre-warmed SDS lysis solution (2% SDS, 16 mM EDTA) and heated to 100°C for 5 minutes. After addition of 1 mL TRIzol, the lysate was incubated for 5 minutes at room temperature, and then frozen at −70°C until RNA was extracted.

RNA extraction was done from four independent biological replicates of each culture at the two sampling time points using the Direct-zol RNA MiniPrep Kit (Zymo Research Co., Irvine, CA). RNA was then precipitated with 2.5 M lithium chloride (Amresco, Solon, OH), re-suspended in nuclease-free water, and treated with DNase I (Invitrogen, Carlsbad, CA) for 30 minutes at 37°C. Ethylenediaminetetraacetic acid (EDTA; 2.3 mM) was then added and the samples were heat-inactivated at 65°C for 10 minutes. RNA quality was assessed using an Agilent 2100 Bioanalyzer (Agilent Technologies, Santa Clara, CA).

cDNA was synthesised from 500 ng of total cellular RNA by random priming using a High Capacity cDNA Reverse Transcription Kit (Applied Biosystems, Foster City, CA) in the presence of RNase inhibitor as per the manufacturer’s instructions.

### Semi-quantitative real-time PCR

Primers were designed using Primer3 as previously described [13], and are listed in Table 2. The amplification efficiencies of all primer pairs were between 95 and 102%. Three technical replicates were done per sample in a total reaction volume of 10 μL, which contained 5 μL of PerfeCTa® SYBR® Green FastMix® (Quanta BioSciences, Inc., Gaithersburg, MD), 2.5 μL of a forward/reverse primer mix with 1.6 μM of each primer, and 2.5 μL of cDNA diluted 1:15.

**Table 2:** **Primers used in this work**

**Primer name**	**Sequence**	**Source**
ASU2-16SrRNA-F1	GTGTAGCGGTGAAATGCGTAGAG	This work
ASU2-16SrRNA-R1	ACATGAGCGTCAGTACATTCCCA	This work
ASU2-ackA-F1	AGCCACCTATTCATCACATCACAA	This work
ASU2-ackA-R1	TACGAACAACAGATACAGAACCACC	This work
ASU2-glyA-F1	GTTTATATCCGAATCCATTACCGCAC	This work
ASU2-glyA-R1	TCATCGCCACAAGCAGAAAGAA	This work
ASU2-gyrA-F1	ATCTGGTATTGCGGTTGGTATGG	This work
ASU2-gyrA-R1	TTCTTCAATGCTGATTTGCTCGTTT	This work
ASU2-proS-F1	GTGGACAAAGCGTCATTACAAGAAAC	This work
ASU2-proS-R1	CGGAAATCTAAACCAAGACGAGTGAA	This work
ASU2-pyk-F1	CAACTGAAGAAGCAATGGACGACA	This work
ASU2-pyk-R1	AACTAACGCAGCATCACCGATTT	This work
ASU2-rho-F1	TTCTTCCTGACGGTTTCGGTTTCTT	This work
ASU2-rho-R1	AACGGCGGATTTGGCTTGGT	This work
ASU2-rpoB-F1	AAACGCAACAAGATCATTCAAGGTG	This work
ASU2-rpoB-R1	ATTTGACGACGAACCGCTAAGTAAA	This work

Real-time PCR was done in a StepOnePlus Thermocycler (Applied Biosystems, Foster City, CA) using a program with an initial denaturation step at 95°C for 30 seconds followed by 40 cycles of 95°C for 3 seconds and 60°C for 30 seconds. Stepwise melt curves were done at the end of each run to confirm that only one template was amplified.

### Reference gene validation

The stability of eight reference genes (Table 1) was assessed using RefFinder (http://www.leonxie.com/referencegene.php), a web-based tool that integrates the algorithms for geNorm, Normfinder, BestKeeper, and the comparative ∆C_t_ methods to rank candidate reference genes from most to least stable. A composite score is then assigned to each gene by taking into account the rankings of the various algorithms employed.

## Results

### Growth curves to determine sampling time points

**Figure 1:**
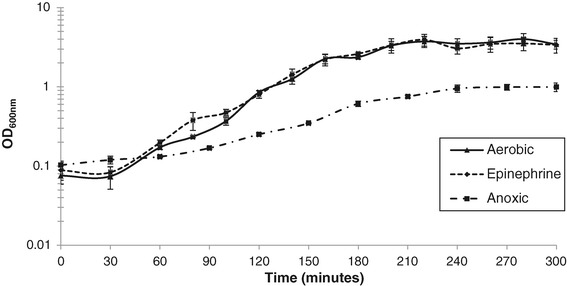
**Growth rates of** ***Actinobacillus suis*** **H91-0380 in BHI in aerobic and anoxic static conditions.** Cultures were grown aerobically with (Epinephrine) and without (Aerobic) 50 μM epinephrine in the growth medium, and under anoxic static (Anoxic) growth conditions.

To determine the times of early exponential and early stationary growth phase, growth curves of aerobic cultures with (Epinephrine) or without (Aerobic) 50 μM epinephrine added to the growth medium at the time of inoculation, and anoxic static cultures (Anoxic) (Figure 1) were done. Aerobic cultures, with or without epinephrine, grew to a higher optical density than the Anoxic cultures. However, the presence of epinephrine in the growth medium did not affect the rate of growth of these cultures relative to Aerobic cultures.

To control for sample variability [14], once the exponential and early stationary growth phase time points were identified, the volume of culture sampled was adjusted to ensure that an approximately equal number of cells was collected for RNA extraction each time; cell numbers were also enumerated by plate counting.

### Reference gene validation based on growth condition and growth phase

Comparisons of comprehensive stability rankings of reference genes were determined for the epinephrine study (Aerobic and Epinephrine samples) and the anoxic study (Aerobic and Anoxic samples). For overall comparison, samples from both the epinephrine and the anoxic studies at each time point (exponential and stationary) and combined samples (collected during both exponential and stationary growth) were also evaluated.

When comparing gene stability for samples collected during exponential growth phase in the epinephrine study (Table 3), the presence of epinephrine in the growth media affected the order of the gene stability rankings. In the Aerobic cultures, *ackA* was the most stably expressed gene, while *glyA* was the least stably expressed. Conversely, in the Epinephrine cultures, *ackA* was the least stably expressed gene and *glyA* was the second most stable gene. In both Aerobic and Epinephrine cultures, *pyk* ranked in the top two most stably expressed genes, so it was not surprising that it was the most stably expressed gene when all samples collected during exponential growth in the epinephrine study were combined. With the exception of *ackA* and *glyA*, the overall order of the stability rankings for the Aerobic and Epinephrine samples was comparable.

**Table 3:** **Comprehensive stability rankings of epinephrine study reference genes from early exponential phase samples**

**Aerobic**		**Epinephrine**		**Combined**	
*ackA*	1.86	*pyk*	1.32	*pyk*	1.19
*pyk*	2.34	*glyA*	2.00	*rpoB*	2.78
*16S rRNA*	2.38	*16S rRNA*	2.71	*ackA*	2.83
*rpoB*	4.05	*rpoB*	4.23	*proS*	3.71
*proS*	4.53	*rho*	4.76	*rho*	4.76
*rho*	4.76	*proS*	5.89	*gyrA*	5.57
*gyrA*	5.83	*gyrA*	5.96	*16S rRNA*	5.73
*glyA*	6.44	*ackA*	6.74	*glyA*	6.44

In the anoxic study, the stability of reference genes in samples collected during exponential growth for both Aerobic and Anoxic cultures were compared (Table 4). The *16S rRNA* gene was the most stably expressed in Anoxic cultures, but it ranked second to last in the Aerobic samples. The second and third most stably expressed genes in the Anoxic samples, *proS* and *gyrA*, ranked in the top three most stably expressed genes in the Aerobic cultures, as well. Interestingly, *rpoB*, the least stably expressed gene in the Aerobic cultures, ranked third in the combined stability rankings of reference genes for exponential samples collected from both Aerobic and Anoxic cultures, while the second most stably expressed gene in the Aerobic samples, *glyA*, was ranked as the least stably expressed gene in the combined scores.

**Table 4:** **Comprehensive stability rankings of anoxic study reference genes from early exponential phase samples**

**Aerobic**		**Anoxic**		**Combined**	
*gyrA*	1.41	*16S rRNA*	1.32	*proS*	1.19
*glyA*	2.11	*proS*	2.21	*gyrA*	1.41
*proS*	2.63	*gyrA*	2.45	*rpoB*	3.41
*pyk*	3.31	*rpoB*	3.50	*16S rRNA*	3.94
*rho*	5.24	*pyk*	4.23	*pyk*	4.95
*ackA*	5.69	*glyA*	6.48	*ackA*	6.09
*16S rRNA*	6.26	*ackA*	6.65	*rho*	6.24
*rpoB*	6.96	*rho*	7.44	*glyA*	8.00

When comparing the stability of reference genes from samples collected in the epinephrine study during stationary phase (Table 5), there was less variation among the top three most stably expressed genes compared to exponential samples. In stationary growth, both *pyk* and *rpoB* ranked in the top three for Aerobic, Epinephrine, and combined samples. The expression of *16S rRNA* was less stable in the Aerobic samples than in the Epinephrine samples, while the opposite was true for *gyrA*. The least stably expressed gene, *ackA*, was consistent in all rankings, and *glyA* was also found in the bottom three rankings for all analyses of these samples.

**Table 5:** **Comprehensive stability rankings of epinephrine study reference genes from early stationary phase samples**

**Aerobic**		**Epinephrine**		**Combined**	
*pyk*	1.50	*16S rRNA*	1.73	*pyk*	1.41
*rpoB*	2.00	*pyk*	2.00	*rpoB*	2.11
*gyrA*	3.22	*rpoB*	2.82	*16S rRNA*	3.76
*rho*	3.31	*proS*	4.23	*proS*	3.83
*proS*	4.56	*rho*	4.30	*rho*	4.30
*16S rRNA*	5.12	*gyrA*	4.56	*glyA*	4.53
*glyA*	5.69	*glyA*	5.23	*gyrA*	5.05
*ackA*	8.00	*ackA*	8.00	*ackA*	8.00

In the samples collected for the anoxic study at stationary phase, the stability rankings differed in Aerobic samples and Anoxic samples (Table 6). The stability scores of *proS*, *16S rRNA,* and *rho* did not differ substantially between these different growth conditions. The scores of *rpoB, pyk, glyA, gyrA,* and *ackA,* however, varied markedly, resulting in very different ranking orders for reference genes measured in the Aerobic and Anoxic cultures.

**Table 6:** **Comprehensive stability rankings of anoxic study reference genes from early stationary phase samples**

**Aerobic**		**Anoxic**		**Combined**	
*rpoB*	1.41	*glyA*	1.41	*pyk*	1.86
*pyk*	1.57	*gyrA*	2.28	*proS*	1.97
*proS*	3.41	*proS*	2.63	*rpoB*	2.06
*gyrA*	4.00	*16S rRNA*	3.94	*gyrA*	4.47
*16S rRNA*	4.14	*rho*	4.76	*glyA*	4.68
*rho*	5.23	*ackA*	5.38	*16S rRNA*	4.76
*glyA*	6.48	*rpoB*	5.69	*ackA*	6.24
*ackA*	8.00	*pyk*	6.96	*rho*	7.24

When the data for all samples collected during exponential and stationary growth in the epinephrine study were analysed together, the overall stability rankings of the top three genes was similar in both growth conditions, and this was reflected in the combined rankings (Table 7), with *proS*, *pyk*, and *glyA* being the most stably expressed. With the exception of *rho*, which consistently ranked as the least stably expressed gene, the difference in stability scores of the remaining 4 reference genes was not substantial.

**Table 7:** **Comprehensive stability rankings of epinephrine study reference genes from exponential and stationary growth phase samples**

**Aerobic**		**Epinephrine**		**Combined**	
*proS*	1.68	*proS*	1.32	*proS*	1.57
*pyk*	1.68	*pyk*	2.06	*pyk*	2.06
*glyA*	2.59	*glyA*	2.63	*glyA*	2.34
*gyrA*	4.12	*16S rRNA*	4.30	*16S rRNA*	4.30
*ackA*	5.05	*rpoB*	4.43	*rpoB*	4.60
*16S rRNA*	5.12	*gyrA*	5.44	*gyrA*	5.23
*rpoB*	5.44	*ackA*	5.73	*ackA*	5.42
*rho*	8.00	*rho*	8.00	*rho*	8.00

Comprehensive rankings of the combined exponential and stationary samples of the anoxic study (Table 8) had similar trends to those observed in the epinephrine study, with *proS* and *pyk* ranking high in stability. The only exception to this was with *glyA* and *gyrA*, where stability in Aerobic and Anoxic cultures differed enough to affect both their stability scores and their overall rankings in each growth condition.

**Table 8:** **Comprehensive stability rankings of anoxic study reference genes from exponential and stationary growth phase samples**

**Aerobic**		**Anoxic**		**Combined**	
*proS*	1.41	*gyrA*	1.97	*proS*	1.41
*glyA*	2.11	*proS*	2.06	*pyk*	2.06
*pyk*	2.91	*pyk*	2.21	*gyrA*	3.22
*ackA*	4.24	*16S rRNA*	3.76	*ackA*	3.98
*gyrA*	4.68	*ackA*	3.81	*glyA*	4.23
*16S rRNA*	4.76	*glyA*	4.90	*16S rRNA*	4.60
*rpoB*	5.69	*rho*	6.74	*rpoB*	6.24
*rho*	7.24	*rpoB*	8.00	*rho*	7.48

Finally, data from samples collected in both the epinephrine and anoxic studies were combined in order to determine overall comprehensive rankings and the effect of multiple growth conditions on gene stability rankings (Table 9). During exponential phase, *16S rRNA, gyrA*, and *ackA* were the most stable reference genes, with comparable stability scores. However, in stationary phase, only *gyrA* remained in the top ranking reference genes for stability, with *16S rRNA* and *ackA* ranking near the bottom of the list. The other two most stably expressed genes were *rpoB*, which ranked 5th for exponential samples, and *pyk*, which ranked 4th. In the combined comprehensive ranking, the most stably expressed reference genes were *pyk, glyA*, and *gyrA*. When comparing these genes to the combined comprehensive rankings from the other analyses, it is interesting to note that at least one of these genes, and sometimes two, ranked among the top three most stably expressed genes in all other conditions. Using geNorm’s calculation of pairwise variation to determine the optimal number of reference genes, it was found that three reference genes were adequate for effective data normalisation (data not shown).

**Table 9:** **Comprehensive stability rankings of genes from epinephrine and anoxic studies at exponential and stationary phase**

**Exponential**		**Stationary**		**Combined**	
*16S rRNA*	2.00	*rpoB*	1.73	*pyk*	2.06
*gyrA*	2.06	*pyk*	1.78	*glyA*	2.38
*ackA*	2.59	*gyrA*	3.22	*gyrA*	2.63
*pyk*	3.81	*glyA*	3.46	*proS*	3.03
*rpoB*	3.94	*proS*	4.70	*rpoB*	4.05
*glyA*	4.74	*ackA*	4.95	*ackA*	4.40
*proS*	5.60	*16S rRNA*	5.29	*16S rRNA*	6.09
*rho*	8.00	*rho*	8.00	*rho*	8.00

In summary, in the epinephrine study, where only aerobic cultures with or without epinephrine in the growth media were compared, two of the same reference genes (*pyk* and *rpoB*) ranked in the top three most stably expressed genes in samples collected during exponential and stationary phases (Tables 3 and 5). However, when the comprehensive stability rankings of all samples in the epinephrine study collected during both phases of growth were compared (Table 7), only *pyk* remained among the top three most stably expressed genes. Similarly, in the anoxic study, where Aerobic and Anoxic samples were grouped together in the experimental design, *proS* and *rpoB* both ranked among the most stably expressed reference genes in the combined rankings for each of the exponential (Table 4) and stationary (Table 6) phase samples, whereas only *proS* ranked among the top three most stably expressed genes of the combined scores for all samples of the anoxic study collected during both growth phases (Table 8).

## Discussion

To date, no studies have been done to characterise reference genes for *A. suis* and our preliminary studies suggested that reference genes used to study closely related organisms were not appropriate. Therefore, the expression stability of eight reference genes was assessed in different growth conditions, growth phases, and with various methods of data analysis. No single reference gene was suitable for normalisation of qPCR results in all growth conditions, sampling time points, or experimental designs. Depending on how the data were analysed, the overall stability rankings of all the reference genes evaluated varied markedly.

Some of the reference genes validated in this study were studied in previous work done by Klitgaard Nielsen and Boye [8] in *Actinobacillus pleuropneumoniae*. Similar to *A. pleuropneumoniae, glyA* and *pyk* were stably expressed in most conditions and time points in *A. suis.* On the other hand, *rho* ranked low in stability in nearly all cases, often at or near the bottom of the list of genes characterised, and demonstrated several C_t_ differences between exponential and stationary phase samples, and between Aerobic and Anoxic samples in the anoxic studies (data not shown).

When choosing appropriate reference genes, consideration should be given to the design of the study, as the number of sampling time points and the different growth phases in which samples are collected can impact the choice of reference genes for data normalisation downstream. Likewise, if the experimental design includes samples from numerous growth conditions with changes in variables such as degree of aeration (shaken vs. static), overall levels of oxygen (aerobic vs. anoxic), different additives in the growth media (presence or absence of epinephrine), and phase of growth when sampling (exponential vs. stationary), the impact on the comprehensive stability rankings of potential reference genes can be drastic. A thorough reference gene validation study of *Staphylococcus epidermidis* by Vandecasteele et al. [15] found that gene expression of purported reference genes, particularly that of *16S rRNA,* varied in response to different growth conditions. In this study, when combining all samples from both the epinephrine study and the anoxic study collected at both exponential and stationary phases (Table 9), *gyrA* was the only reference gene that ranked among the top three most stably expressed genes during exponential phase, stationary phase, and combined sampling time points.

The determination of whether to keep or discard a reference gene can be made based on the stability scores assigned by the different algorithms that go into determining the composite score. If a gene is found to rank consistently low in stability by most or all of the individual algorithms for a given experimental design, the composite score of this gene will reflect this due to the weighted calculation employed in its determination. Similarly, the cut-off between a suitable or unsuitable reference gene can be considered in the context of the individual algorithms depending on the design of the study. BestKeeper and geNorm employ similar techniques of pairwise comparisons of reference genes while considering the dataset as a whole rather than considering the possible effects of comparing different time points or replicates collected [16]. On the other hand, NormFinder takes into account these latter types of variation, and compares each gene to the mean derived from the dataset and so it is better able to identify the gene(s) with the greatest stability in the conditions included in the dataset. To benefit from the strengths of each algorithm, and to limit the inherent biases from the assumptions employed by these different methods of reference gene validation, the composite score assigned by RefFinder reflects the geometric mean of the weighted ranking of a gene from the different algorithms. It is also valuable to employ geNorm’s calculation of pairwise variations in normalisation factors for different combinations of reference genes in order to determine the optimum number of reference genes recommended for accurate normalisation [7]. Employing this method in addition to the stability rankings from the algorithms and the composite scores from RefFinder allows for a reasonable validation of the most stably expressed reference genes as well as the ideal number of reference genes suited to a given experimental design based on the genes tested.

There have been few reference gene validation studies published for members of the family *Pasteurellaceae*. In two studies of *Haemophilus ducreyi*, qPCR was used to validate expression of a subset of genes from RNA-Seq or microarray findings. In the RNA-Seq study [17], *dnaE* was used to normalise qPCR results, but no mention was made as to why this gene was chosen or if its stability was validated by qPCR. In the microarray study [18], *gyrB* was used to normalise the cDNA per sample because transcript levels of this gene did not change during DNA microarray experiments. In a study of the expression of *Pasteurella multocida* virulence genes during experimental infection of mice, *16S rRNA* was used to normalise qPCR results [9], but it was not explicitly stated why this gene was chosen or if it was validated for this study. In a study of *A. pleuropneumoniae* biofilms cultured under static and planktonic conditions and sampled at different time points, qPCR was used to validate microarray results [19]. In this study, the results were normalised using *rlu*C based on its constant signal in the microarrays, though it is not clear whether this gene was also validated independently by qPCR. In a study of *Aggregatibacter actinomycetemcomitans*, qPCR was used to look at whether the expression of selected genes from *in vivo*-induced antigen technology in human infections was consistent with the expression of the same genes during epithelial cell interaction. In this work, *16S rRNA* and *gapdh* were used for normalisation [20]. While these reference genes were validated, the authors mentioned that their expression was variable and that they were differentially expressed under experimental conditions. Despite this, Longo et al. [21] used *gapdh* to normalise expression data in a later study. Finally, in a study looking at gene expression of *Mannheimia haemolytica* at two time points during experimental infection of calves and at early log phase of bacteria grown *in vitro, dnaN* was used to normalise qPCR results based on its apparent stable expression in a previous microarray study [22]; however, no mention was made as to whether this gene was specifically validated for qPCR.

There have been many studies where *16S rRNA* has been used as the sole reference gene for data normalisation with little or no data provided regarding its suitability. Others have observed variability in the stability of *16S rRNA* for various organisms grown under different conditions, and even strain-to-strain variation among members of the same species [15,23]. In the current study, *16S rRNA* ranked as the most stably expressed gene in three instances (Tables 4, 5, and 9), and in the top three most stably expressed genes for combined data once (Table 5). This is not to say that *16S rRNA* is not suitable for some studies, but caution should be taken in assuming that this gene is stably expressed in all growth conditions and growth phases, and its suitability should be assessed for each study and experimental design.

## Conclusions

The current study demonstrated the relative stability rankings of eight reference genes in *A. suis* in different growth conditions and growth phases. This work lends further support to the notion that reference genes must be carefully assessed with all of the experimental conditions in mind, and that one should not rely on commonly used genes without first demonstrating the stability of their expression under the specific conditions under study. As more qPCR studies are reported, it may be possible to make informed predictions as to which reference genes might be useful to select for validation studies; however, in the meantime, caution is warranted. Finally, these data support the recommendations of Vandesompele et al. [7] that the best approach to normalise qPCR results may be to use the geometric mean of multiple reference genes.
